# OxLDL-induced ferroptosis and pyroptosis in atherosclerosis: a mini review

**DOI:** 10.3389/fimmu.2026.1831457

**Published:** 2026-05-28

**Authors:** Shaozhi Liu, Yuxia Wu, Lei Chen, Li Hu, Jinjin Yin

**Affiliations:** 1 Department of Pharmacy, Guangdong Provincial Key Laboratory of Major Obstetric Diseases, Guangdong Provincial Clinical Research Center for Obstetrics and Gynecology, The Third Affiliated Hospital, Guangzhou Medical University, Guangzhou, China; 2 Hainan General Hospital, Hainan Affiliated Hospital of Hainan Medical University, Haikou, Hainan, China; 3 Department of Hepatology, The First Hospital of Hunan University of Chinese Medicine, Changsha, China

**Keywords:** atherosclerosis, ferroptosis, gasdermin D, lipid peroxidation, NLRP3 inflammasome, oxidized low-density lipoprotein, pyroptosis

## Abstract

Oxidized low-density lipoprotein (oxLDL) is a central driver of inflammatory responses in atherosclerosis and triggers multiple forms of regulated cell death beyond classical apoptosis. Ferroptosis, characterized by iron-dependent lipid peroxidation (LPO), and pyroptosis, mediated by inflammasome-activated gasdermin D (GSDMD) pore formation, have emerged as critical contributors to plaque progression and instability. Recent evidence highlights a significant crosstalk between these two death modalities: the N-terminal fragment of GSDMD targets mitochondrial membranes to promote LPO, while ferroptotic byproducts—including oxidized phospholipids and 4-hydroxynonenal—activate the NOD-like receptor family pyrin domain containing 3 (NLRP3) inflammasome. This bidirectional interplay establishes a positive feedback loop that amplifies vascular inflammation. This review summarizes the molecular mechanisms underlying oxLDL-induced ferroptosis and pyroptosis, emphasizes their interconnected regulatory networks, and discusses therapeutic strategies targeting this cell death axis. Understanding this integrated cell death network may provide new insights for resolving residual inflammatory risk in atherosclerotic cardiovascular disease.

## 1 Introduction

Despite significant advances in lipid-lowering therapy, atherosclerotic cardiovascular disease (ASCVD) remains the leading cause of mortality worldwide, accounting for approximately 18.6 million deaths annually (1). Even when low-density lipoprotein cholesterol (LDL-C) is optimally controlled, a persistent residual cardiovascular risk underscores the necessity for therapeutic approaches targeting inflammatory pathways (2). The CANTOS trial provided clinical validation for the inflammation hypothesis in atherosclerosis by demonstrating that interleukin-1β (IL-1β) inhibition reduces major adverse cardiovascular events independently of lipid-lowering effects (3).

Ox-LDL is a key pathogenic factor in atherogenesis. Native LDL undergoes oxidative modification after retention in the subendothelial space, generating various bioactive lipids, including oxidized phospholipids, lipid hydroperoxides, 4-hydroxynonenal (4-HNE), and malondialdehyde (MDA) (4, 5). These oxidized lipids promote foam cell formation, endothelial dysfunction, and inflammatory activation through scavenger receptor-mediated uptake and pattern recognition receptor signaling (6, 7).

While apoptosis has traditionally been considered the predominant form of cell death in atherosclerotic lesions, emerging evidence indicates that oxLDL can also trigger ferroptosis and pyroptosis—two distinct regulated cell death modalities with unique mechanisms and inflammatory consequences (8, 9). Ferroptosis proceeds through iron-catalyzed LPO and is suppressed by glutathione peroxidase 4 (GPX4) (10). Pyroptosis is mediated by inflammasome-activated caspase-1, which cleaves GSDMD, leading to pore formation and release of inflammatory cytokines (11, 12). Importantly, recent studies have revealed extensive molecular crosstalk between these pathways (13, 14), suggesting that they constitute an integrated cell death network in atherosclerosis. This review focuses on the analysis of oxLDL-induced ferroptosis and pyroptosis, emphasizing their molecular mechanisms, bidirectional interactions, and therapeutic implications.

## 2 OxLDL-induced ferroptosis

Ferroptosis is an iron-dependent, LPO-driven form of programmed cell death, characterized by the uncontrolled accumulation of reactive oxygen species (ROS) in membrane lipids following GPX4 inactivation (15). In the pathological progression of atherosclerosis, oxLDL systematically promotes ferroptosis in vascular cells through multiple convergent mechanisms targeting iron homeostasis, antioxidant defense systems, and membrane lipid composition, thereby influencing plaque stability and disease progression.

### 2.1 Molecular mechanisms of ferroptosis induction

#### 2.1.1 Ferroptosis-specific biomarker

Ferroptosis is an iron-dependent form of regulated cell death driven by membrane LPO. Its core mechanisms involve three interconnected processes: dysregulated iron metabolism, antioxidant system failure, and LPO (16). In iron metabolism, extracellular Fe³^+^ is internalized via transferrin receptor-mediated endocytosis, reduced to Fe²^+^, and transported into the cytoplasm by Divalent Metal Transporter 1 (DMT1). The free iron then generates ROS through the Fenton reaction (17). In antioxidant defense, System Xc^-^ (SLC3A2/SLC7A11) exchanges extracellular cystine for intracellular glutamate; the imported cystine is reduced to cysteine for glutathione (GSH) synthesis, which maintains GPX4 activity to eliminate lipid peroxides (16, 18, 19). Inhibition of this pathway depletes GSH and suppresses GPX4, leading to lipoxygenase-driven accumulation of lipid peroxides and ultimately ferroptosis (16). Thus, specific biomarkers of ferroptosis include reduced GPX4 expression/activity, decreased GSH levels, elevated LPO products (e.g., 4-HNE, MDA), and intracellular free iron accumulation, serving as core indicators for detecting and assessing ferroptosis.

#### 2.1.2 OxLDL uptake and initiation of LPO

Scavenger receptor-mediated oxLDL uptake represents a critical initiating event for ferroptosis. OxLDL is primarily internalized through CD36 and lectin-like oxidized low-density lipoprotein receptor-1 (LOX-1), delivering oxidized lipids to cellular membranes and initiating peroxidative chain reactions (20, 21). Within atherosclerotic plaques, the accumulation of oxLDL-induced lipid peroxides constitutes a primary condition for ferroptosis occurrence. Iron overload, together with oxLDL serving as the main substrate for LPO, jointly constitutes key factors promoting ferroptosis and atherosclerosis progression (22).

#### 2.1.3 Dysregulation of iron homeostasis

Iron homeostasis dysregulation is a central mechanism in oxLDL-induced ferroptosis. OxLDL promotes iron uptake by upregulating transferrin receptor 1 (TfR1) expression while inhibiting ferritin heavy chain (FTH1) and ferroportin expression, thereby impairing iron storage and efflux and leading to significant expansion of the labile iron pool (10, 23–27). Macrophages within atherosclerotic plaques exhibit markedly elevated intracellular free iron levels, and iron chelation therapy with deferoxamine (DFO) effectively attenuates atherosclerotic lesions in experimental models (28).

#### 2.1.4 Impairment of antioxidant defense systems

**Figure 1:**
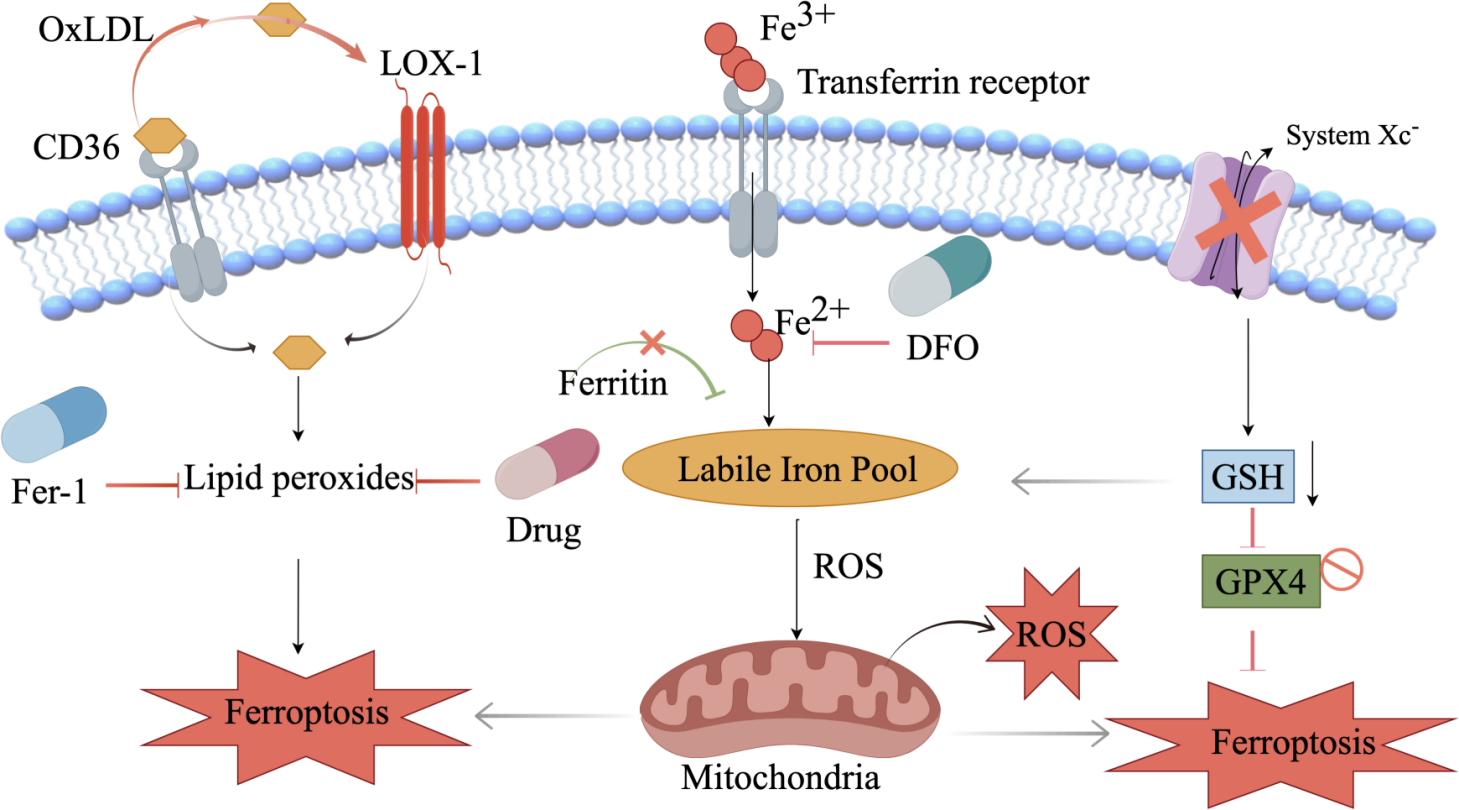
Molecular mechanisms of OxLDL-induced ferroptosis in atherosclerosis. OxLDL, oxidized low-density lipoprotein; LOX-1, lectin-like oxidized LDL receptor-1; CD36, cluster of differentiation 36; Fe^3+^,Ferric ion; Fe²^+^, ferrous iron; DFO, deferoxamine; Fer-1, Ferrostatin-1; ROS, reactive oxygen species; GSH, glutathione; GPX4, glutathione peroxidase 4; System Xc^-^, cystine/glutamate antiporter.

Multilevel impairment of antioxidant defense systems by oxLDL is a critical determinant of ferroptosis susceptibility. First, oxLDL reduces cysteine uptake by inhibiting SLC7A11 (the cystine/glutamate antiporter, System Xc-), thereby compromising GSH synthesis and exacerbating oxidative stress imbalance (29). Second, oxLDL suppresses nuclear factor erythroid 2-related factor 2 (Nrf2) activity, reducing the transcriptional expression of GPX4 and System Xc- components (30, 31) (shown in **Figure 1**).

GPX4, as the core negative regulator of ferroptosis, suppresses ferroptosis by eliminating lipid peroxides, and its inactivation is a key event in ferroptosis occurrence (18). The oxLDL-associated LPO product 4-HNE can directly modify GPX4 (shown in **Figure 1**), reducing its enzymatic activity and stability, thereby compromising lipid peroxide clearance capacity and amplifying LPO cascades (22, 32, 33). Studies have confirmed that oxLDL induces ferroptosis through the dual action of iron accumulation and GPX4 inhibition, resulting in LPO and endothelial dysfunction (34).

#### 2.1.5 Pharmacological interventions in ferroptosis

The 4-HNE-GPX4 axis-mediated LPO/ferroptosis phenotype can be suppressed by various pharmacological approaches. Iron chelators reduce the labile iron pool, while lipophilic radical-trapping antioxidants block LPO chain reactions, both effectively inhibiting ferroptosis. Additionally, enhancing GPX4 expression significantly reduces cellular sensitivity to ferroptosis (18, 22, 35). In mouse aortic endothelial cells (MAECs), oxLDL or erastin treatment leads to significantly elevated ROS, lipid peroxides, and MDA levels in damaged mitochondria, whereas Fer-1 effectively suppresses the generation of these peroxidation products, indicating that ferroptosis inhibition ameliorates oxLDL-induced endothelial cell injury (34), shown in **Figure 1**.

### 2.2 Cell type-specific ferroptosis

Different vascular cells exhibit differential susceptibility to ferroptosis, and this cell type specificity plays important roles at various stages of atherosclerosis.

#### 2.2.1 Endothelial cells

Endothelial dysfunction is the initiating event in atherosclerosis. Endothelial cells highly express LOX-1 and are particularly sensitive to oxLDL-mediated LPO (24). Endothelial ferroptosis leads to barrier dysfunction, increased permeability, and reduced nitric oxide bioavailability, promoting early atherogenic events (36). Single-cell RNA sequencing has identified ferroptosis-related gene signatures in endothelial cell clusters from human atherosclerotic plaques (37).

#### 2.2.2 Macrophages and foam cells

Macrophages transform into foam cells after extensive oxLDL phagocytosis during plaque formation, with concomitant aberrant intracellular iron accumulation. When iron overload exceeds cellular antioxidant defense capacity, lipid peroxides accumulate massively, triggering macrophage ferroptosis (28, 34). Foam cell ferroptosis releases LPO products and iron into the extracellular environment, propagating oxidative damage to neighboring cells (18). Furthermore, iron metabolism dysregulation in macrophages participates in inflammatory responses, exacerbating atherosclerosis severity (38).

#### 2.2.3 Vascular smooth muscle cells

Vascular smooth muscle cell (VSMC) ferroptosis directly contributes to fibrous cap thinning, affecting plaque structural stability. Studies have shown that VSMC-specific GPX4 overexpression significantly increases fibrous cap thickness in ApoE^-^/^-^ mice (39, 40), suggesting that targeting VSMC ferroptosis may be an effective strategy for plaque stabilization.

### 2.3 Ferroptosis and plaque vulnerability

Vulnerable plaques are characterized by a large lipid-rich necrotic core, a thin fibrous cap, and active inflammation. Ferroptosis plays an important role in each of these three key features.

#### 2.3.1 Necrotic core expansion

Accumulation of LPO products within plaques correlates closely with necrotic core expansion (41). Dying foam cells release lipid debris and pro-inflammatory factors, further enlarging the necrotic core and forming a vicious cycle (42).

Ferroptosis exhibits distinct morphological features that differentiate it from apoptosis and necrosis. Unlike apoptotic cells, which undergo cell shrinkage, membrane blebbing, and nuclear condensation while maintaining membrane integrity until late stages, ferroptotic cells preserve normal nuclear morphology and rounded cell shape but display characteristic mitochondrial alterations—including mitochondrial shrinkage, increased membrane density, loss of cristae, and outer membrane rupture—accompanied by compromised plasma membrane integrity (10, 17). These distinct features underlie the unique mechanism by which ferroptosis, rather than apoptosis alone, drives necrotic core expansion in atherosclerotic plaques.

While ox-LDL can induce both apoptosis and ferroptosis in macrophages, ferroptosis uniquely propagates cell death through a self-amplifying cascade. When macrophages undergo ferroptosis, the rupture of their plasma membrane releases labile iron and lipid peroxidation debris into the plaque microenvironment (23–27). The released iron is subsequently taken up by neighboring macrophages via TfR1, expanding their labile iron pool and rendering them susceptible to ferroptosis (41). ox-LDL further intensifies this iron-driven propagation by upregulating TfR1 and acyl-CoA synthetase long-chain family member 4 (ACSL4) in vascular cells, thereby promoting lipid peroxidation and ferroptotic injury (42). Concurrently, ox-LDL activates the NLRP3 inflammasome in macrophages, amplifying local inflammation and ferroptosis (43), which further aggravates atherosclerotic progression (34).

Bai et al. demonstrated that ferrostatin-1 (Fer-1) simultaneously acts on all three core ferroptosis pathways in HFD-fed ApoE^-^/^-^ mice: it reduced iron accumulation in serum and aortic tissues, suppressed lipid peroxidation (evidenced by decreased MDA and LPO levels), and restored antioxidant defenses (by upregulating SLC7A11 and GPX4), collectively attenuating atherosclerotic lesion progression (34). Ferroptosis inhibition also effectively prevented ox-LDL-induced lipid peroxidation and endothelial dysfunction in mouse aortic endothelial cells (MAECs) (34).

#### 2.3.2 Fibrous cap thinning

Smooth muscle cells, as the key cells responsible for collagen synthesis and maintenance of fibrous cap structural integrity, are also threatened by ferroptosis. Iron overload and oxidative stress in the plaque microenvironment can induce smooth muscle cell ferroptosis, leading to decreased collagen synthesis capacity and progressive fibrous cap thinning, significantly increasing the risk of plaque rupture (44).

#### 2.3.3 Intraplaque hemorrhage and iron release

Advanced plaques often contain neovascularization, and these vessels are structurally fragile and prone to rupture and hemorrhage. Erythrocyte lysis releases substantial heme-derived free iron, further exacerbating local ferroptosis and oxidative damage (45).

#### 2.3.4 Amplification of inflammatory cascades

Damage-associated molecular patterns (DAMPs) released during ferroptosis activate innate immune responses, recruiting more monocytes and neutrophils to infiltrate plaques and continuously amplifying inflammatory cascades. This ultimately forms a “ferroptosis-inflammation-plaque instability” vicious cycle, greatly increasing the risk of acute cardiovascular events. Decreased GPX4 activity and iron metabolism dysregulation are key mechanisms in this pathological process, and targeting ferroptosis pathways represents a promising new strategy for preventing plaque rupture (34).

## 3 OxLDL-induced pyroptosis

Pyroptosis is a form of inflammatory programmed cell death triggered by various stimuli. Its molecular hallmarks include inflammasome assembly and activation, plasma membrane pore formation, and maturation and release of pro-inflammatory cytokines. Based on whether caspase-1 activation is required, pyroptosis can be classified into canonical inflammasome pathways and non-canonical inflammasome pathways (46). In the pathological progression of atherosclerosis, oxLDL activates the NLRP3 inflammasome through multiple mechanisms, triggering GSDMD-mediated pyroptosis, releasing pro-inflammatory factors, and continuously amplifying inflammatory responses within plaques.

### 3.1 Specific biomarkers for pyroptosis

Pyroptosis, an inflammatory cellular death, is accompanied by cellular oedema, plasma membrane pore formation, causing the release of proinflammatory cytoplasmic molecules containing interleukin (IL)‐1β and IL‐18 (47). Mechanically, canonical caspase‐1 activation upon the NLRP3 inflammasome cleaves GSDMD into its active form, amino‐terminal GSDMD (48). The N‐terminal GSDMD‐N domain, a pore‐drilling fragment, results in the perforation on membrane and ultimately causes pyroptosis (49). Therefore, the identification of pyroptosis relies on the core biomarker axis consisting of caspase-1 activation, Gasdermin D N-terminal fragment (GSDMD-NT) mediated membrane pore formation, and the release of mature IL-1β and IL-18.

### 3.2 Mechanisms of NLRP3 inflammasome activation

The NLRP3 inflammasome plays a pivotal role in the progression of atherosclerosis. OxLDL activates macrophage TLR2/TLR4 (50–52) and upregulates the TLR4/NF-κB signaling pathway (53, 54), thereby promoting monocyte-to-foam cell transformation (55) and providing the priming signal for NLRP3 inflammasome activation. NLRP3 is highly expressed in macrophage-derived foam cells within atherosclerotic plaques (56), where it recruits ASC to activate caspase-1, subsequently driving the maturation and secretion of IL-1β/IL-18 and triggering pyroptosis (57–60). Deficiency of TLR2/TLR4 reduces lipid accumulation in plaques (53, 61), and inhibition of the TLR4/NF-κB/NLRP3 axis attenuates oxLDL-induced foam cell pyroptosis (54), highlighting this pathway as a critical therapeutic target for inflammatory injury in atherosclerosis.

#### 3.2.1 ROS and mitochondrial damage pathways

ROS generated by oxLDL promote NLRP3 activation through oxidative modification of NLRP3 protein and activation of thioredoxin-interacting protein (TXNIP) (62, 63). Mitochondrial damage induced by various cellular stresses can lead to the release of oxidized mitochondrial DNA (mtDNA) into the cytoplasm, serving as a key NLRP3 activation signal (64). In the atherosclerotic pathological environment, oxLDL is one of the main stressors inducing such mitochondrial damage and oxidized mtDNA release in macrophages.

Cellular autophagy, particularly mitophagy which clears damaged mitochondria, is crucial for suppressing this process and preventing excessive NLRP3 activation (65). When autophagy is impaired, damaged mitochondria accumulate, and oxidized mtDNA is continuously released, leading to sustained NLRP3 inflammasome activation.

#### 3.2.2 Endoplasmic reticulum stress pathway

Endoplasmic reticulum (ER) stress represents another important pathway for NLRP3 inflammasome activation. During atherosclerosis progression, oxLDL accumulation in macrophages can trigger the unfolded protein response (UPR), wherein the IRE1α signaling pathway promotes TXNIP upregulation. TXNIP subsequently binds directly to NLRP3, leading to inflammasome activation and programmed cell death (66). Consistent with this, alleviating macrophage ER stress using chemical chaperones inhibits NLRP3 activation and attenuates atherosclerotic lesions in experimental models (67).

### 3.3 GSDMD-mediated pyroptosis execution

Following NLRP3 inflammasome activation, a multiprotein complex composed of NLRP3, ASC, and pro-caspase-1 assembles, promoting caspase-1 self-cleavage and activation. Activated caspase-1 performs dual functions: it cleaves inactive pro-IL-1β and pro-IL-18 precursors, converting them into mature inflammatory cytokines, and it cleaves GSDMD, generating a pore-forming GSDMD-NT by releasing the autoinhibitory Gasdermin D C-terminal fragment (GSDMD-CT) (11, 68–70).

The released GSDMD-NT translocates to the plasma membrane, binds to membrane phospholipids, and oligomerizes to form transmembrane pores with an inner diameter of approximately 10–20 nm (71). These membrane pores have dual pathological effects: they allow the release of inflammatory cytokines such as IL-1β and IL-18 into the extracellular environment (72), and they cause osmotic imbalance, cell swelling, and ultimately pyroptotic cell death (68–70).

#### 3.3.1 Pathological roles of pyroptosis-released factors

IL-1β, as a key inflammatory mediator released during pyroptosis, promotes endothelial activation, monocyte recruitment, and vascular smooth muscle cell (VSMC) proliferation, serving as a core driver of atherosclerosis progression (73). Clinical studies indicate that elevated IL-18 levels are strong predictors of cardiovascular death in patients with stable and unstable angina (74).

#### 3.3.2 GSDMD expression in atherosclerosis

Compared with normal arteries, human atherosclerotic plaques exhibit significantly elevated GSDMD expression, with particularly high levels in advanced lesions, suggesting that pyroptosis plays an important role in plaque progression and instability (69). This finding provides a theoretical basis for therapeutic strategies targeting GSDMD.

### 3.4 Non-canonical pyroptosis pathways

In addition to the NLRP3-caspase-1 canonical pyroptosis pathway, non-canonical pyroptosis pathways are also activated in atherosclerosis.

#### 3.4.1 LPS-caspase-4/5/11 pathway

The mechanistic basis of non-canonical pyroptosis is that intracellular lipopolysaccharide (LPS) derived from the gut microbiota or other sources can directly bind and activate caspase-4/5 (in humans) or caspase-11 (in mice). These activated caspases can directly cleave GSDMD independently of canonical inflammasome complexes, inducing pyroptosis (75, 76).

In the atherosclerotic microenvironment, factors such as oxLDL may promote LPS entry into the cytoplasm by disrupting cell membrane stability or increasing endocytosis, potentially triggering this non-canonical pathway. This pathway may be particularly relevant in patients with metabolic syndrome, where intestinal barrier dysfunction increases circulating LPS levels, providing a pathological basis for non-canonical pyroptosis pathway activation (77).

#### 3.4.2 TAK1 inhibition-caspase-8 pathway

Additionally, when TAK1, a key inflammatory signaling kinase, is inhibited, a caspase-8-mediated GSDMD cleavage pathway is initiated independently of canonical inflammasomes, leading to cell death (78). The existence of this alternative pathway suggests that the regulatory network of pyroptosis is more complex than initially appreciated, providing new perspectives on the activation mechanisms of pyroptosis in atherosclerosis.

## 4 Crosstalk between ferroptosis and pyroptosis

### 4.1 Shared upstream signals

Oxidative stress serves as a key hub connecting ferroptosis and pyroptosis. ROS play a dual role in this context: ROS can induce the expression and activation of thioredoxin-interacting protein (TXNIP), which subsequently binds and activates the NLRP3 inflammasome, initiating the caspase-1/GSDMD-dependent canonical pyroptosis pathway (62). More critically, ROS-triggered membrane phospholipid peroxidation not only constitutes the execution basis of ferroptosis but also generates specific LPO products that can directly modify and activate the pyroptosis execution protein GSDMD, leading to pyroptosis (79). In the context of oxLDL stimulation in atherosclerosis, these dual ROS-driven mechanisms may act simultaneously or synergistically to exacerbate vascular cell death and inflammation.

In atherosclerosis, oxLDL stimulation can lead to mitochondrial dysfunction in vascular cells. This process may simultaneously drive ferroptosis and pyroptosis, forming a synergistic death-inflammation axis: damaged mitochondria undergo LPO events such as cardiolipin oxidation, which is a core execution event in ferroptosis (80–82), while damaged mitochondria release oxidized mitochondrial DNA (mtDNA), a potent activator of the NLRP3 inflammasome (65). Thus, oxLDL-induced mitochondrial damage may diverge into different downstream pathways (LPO vs. mtDNA release) through shared upstream events (mitochondrial dysfunction), thereby simultaneously or synergistically exacerbating lipotoxic death (ferroptosis) and inflammatory death (pyroptosis) in vascular cells, collectively promoting plaque instability.

Cholesterol crystals can induce lysosomal rupture, activating the NLRP3 inflammasome, a key mechanism in atherosclerosis development (83). Cathepsin B released after lysosomal damage is an important mediator of NLRP3 activation (84). In the context of atherosclerosis, cholesterol crystal-induced lysosomal rupture may release free iron. Studies have confirmed that lysosomes are an important source of free iron required for ferroptosis (85, 86), suggesting that lysosomal damage may simultaneously trigger both pyroptosis and ferroptosis, further indicating that cholesterol crystals may induce both forms of cell death.

### 4.2 GSDMD promotes LPO

Upon activation of gasdermin family proteins (particularly GSDMD), the GSDMD-NT oligomerizes to form pores in the plasma membrane, initiating pyroptosis. This process leads to the loss of intracellular small molecules, including ions and GSH. GSH depletion impairs GPX4 activity, compromising the cellular capacity to eliminate lipid peroxides and thereby driving ferroptosis occurrence (79). Furthermore, GSDME, another family member, when activated, its N-terminal fragment can target mitochondrial membranes to form pores, leading to mitochondrial dysfunction, ROS burst, and promotion of LPO, thereby amplifying cell death signals (87). Additional studies have shown that GSDMD pores facilitate iron influx, further promoting the ferroptotic process (88). This integrated effect positions GSDMD as a molecular amplifier of ferroptosis under inflammatory conditions. Pharmacological inhibition of GSDMD with disulfiram reduces both IL-1β release and LPO in oxLDL-treated macrophages (89). These findings reveal profound mechanistic intersections between different programmed cell death modalities, providing new potential therapeutic targets for related diseases.

### 4.3 Ferroptosis products activate NLRP3

Ferroptosis amplifies pyroptosis through the release of immunostimulatory DAMPs. DAMPs can induce the expression of components such as the inflammasome NLRP3 (72, 90, 91). Oxidized cardiolipin released from damaged mitochondria binds to NLRP3 and promotes inflammasome assembly (92). Ferroptosis is an iron-dependent, LPO-driven form of cell death. Studies have shown that lipid peroxides and their metabolites (such as 4-hydroxynonenal, 4-HNE) and oxidized phospholipids generated during ferroptosis can serve as DAMPs, activating the NLRP3 inflammasome. Furthermore, free iron released during ferroptosis promotes ROS generation, further enhancing NLRP3 assembly and activation, leading to maturation and secretion of IL-1β and IL-18, thereby amplifying inflammatory responses (26, 93, 94).

### 4.4 Cytokine-mediated ferroptosis sensitization

Ferroptosis releases oxidized phospholipids/lipid peroxides and iron ions, inducing mitochondrial ROS and promoting NLRP3 assembly, activating caspase-1 to mature IL-1β/IL-18, driving pyroptotic inflammatory amplification (95, 96).

### 4.5 The effects on innate immune system ferroptosis and pyroptosis in atherosclerosis

Ferroptosis and pyroptosis exert significant side effects on the innate immune system, thereby exacerbating atherosclerotic inflammation. Ferroptotic cells release various DAMPs, including HMGB1, ATP, and oxidized phospholipids, which activate pattern recognition receptors such as TLRs and RAGE on macrophages, neutrophils, and dendritic cells, amplifying sterile inflammation within plaques (41). These DAMPs drive macrophage polarization toward a pro-inflammatory M1 phenotype, resulting in excessive production of TNF-α, IL-6, and IL-1β. Additionally, labile iron released from ferroptotic cells is taken up by surrounding macrophages, increasing their iron burden and rendering them more susceptible to ferroptosis, thus establishing a self-reinforcing cycle of innate immune cell death and inflammation (97). Similarly, OxLDL-induced pyroptosis leads to the formation of gasdermin D pores, allowing uncontrolled release of DAMPs and the potent pro-inflammatory cytokines IL-1β and IL-18 (11). These secreted mediators activate neighboring macrophages and promote their polarization toward a pro-atherogenic M1 phenotype, amplifying local vascular inflammation. Moreover, IL-1β and other chemokines released during pyroptosis recruit circulating neutrophils into the plaque, where they can undergo NETosis, releasing neutrophil extracellular traps (NETs) that further activate macrophages and endothelial cells via Toll-like receptors. Importantly, NETs themselves can trigger NLRP3 inflammasome activation in macrophages, establishing a positive feedback loop that perpetuates pyroptosis and sustains innate immune activation (98). Collectively, these side effects demonstrate that both ferroptosis and pyroptosis are not merely modes of cell death but potent immunostimulatory events that sustain and amplify innate immune-driven chronic inflammation in atherosclerosis.

## 5 Therapeutic implications

The therapeutic strategies discussed above, targeting ferroptosis, pyroptosis, or their crosstalk, are summarized in **Table 1**. This overview highlights the diverse mechanisms of action, current stages of development, and key evidence supporting each approach, providing a comprehensive reference for future research and clinical translation.

**Table 1:** Therapeutic strategies targeting ferroptosis and pyroptosis in atherosclerosis.

Target	Intervention	Mechanism of Action	Stage of Development	Key Findings/References
Ferroptosis	Ferrostatin-1	Lipid peroxidation inhibitor	Preclinical	Reduces plaque area and necrotic core in ApoE^-^/^-^ mice (99)
	Liproxstatin-1	Inhibits ferroptosis by decreasing VDAC1 levels and restoring GPX4 expression, thereby reducing lipid peroxidation and mitochondrial dysfunction	Preclinical	Liproxstatin-1 protects mouse myocardium against ischemia/reperfusion injury, improves cardiac function recovery, and reduces infarct size (100)
	Selenium supplementation	Enhances GPX4 synthesis and activity	Clinical observation	Prevents ferroptosis in cell models (101)
	Vitamin E	Endogenous antioxidant; Eliminating oxygen free radicals, it can also competitively bind to and inhibit LOXs	preclinical/established research	Prevent ferroptosis (103)
	Coenzyme Q10	The FSP1-CoQ10 system represents a parallel, GPX4-independent pathway for preventing ferroptosis	Preclinical research	May provide additional protection for cells against LPO and ferroptosis (104)
NLRP3 inflammasome	MCC950	Selective NLRP3 inhibitor	Animal experiments	Reduces plaque progression and improves stability (105)
	Colchicine	Inhibits NLRP3 assembly and activation	Clinical (approved for CAD)	31% MACE reduction (108)
	Canakinumab	IL-1β monoclonal antibody	Clinical (CANTOS trial)	Reduces MACE independent of lipid lowering (3)
GSDMD	Disulfiram	Covalently modifies GSDMD, blocks pore formation	Ex vivo experiment	Reduces IL-1β release and lipid peroxidation in vitro (89)
	Necrosulfonamide	GSDMD inhibitor	Ex vivo experiment	Inhibits pyroptotic cell death (110)
Dual/multi-target	Statins	Lipid-lowering + NLRP3 inhibition	Clinical	Anti-inflammatory effects beyond LDL reduction (112, 113)

The table summarizes key interventions targeting ferroptosis, NLRP3 inflammasome, GSDMD, or multiple pathways simultaneously. For each intervention, the mechanism of action, current stage of development, and representative findings with corresponding references are provided. CAD, coronary artery disease; GSDMD, gasdermin D; IL-1β, interleukin-1β; MACE, major adverse cardiovascular events; NLRP3, NLR family pyrin domain containing 3; FSP1, ferroptosis suppressor protein 1;VDAC, voltage-dependent anion channel; LOXs, lipoxygenases; CoQ10,coenzyme Q10.

### 5.1 Targeting ferroptosis

Ferroptosis inhibitors exert their effects by either suppressing LPO or strengthening antioxidant defense systems. To date, several well-characterized ferroptosis inhibitors have shown protective effects against ferroptosis-related cardiovascular diseases (CVDs), the most thoroughly studied iron death inhibitor is the free radical scavenging antioxidant (RTA) (9). Preclinical models have demonstrated that the lipophilic radical-trapping antioxidants Fer-1 and Liproxstatin-1 significantly reduce plaque area and necrotic core size in ApoE^-^/^-^ mice by effectively inhibiting LPO cascades (99), and by restoring the content of GPX4, the ischemia/reperfusion injury of the mouse myocardium can be alleviated (100). The emergence of various iron-regulating therapies highlights the importance of maintaining iron homeostasis in the cardiovascular system. However, many iron-based therapies, such as intravenous iron supplementation, may produce adverse effects, and future research needs to identify patient populations with iron deficiency (ID) who would truly benefit from treatment (101). Additionally, enhancing endogenous antioxidant defenses represents another important direction: selenium supplementation prevents ferroptosis in cell models by ensuring GPX4 synthesis and activity (102), while vitamin E, as a classic lipophilic antioxidant, not only can it detect oxygen free radicals, but it can also prevent ferroptosis by competitively binding and inhibiting lipoxygenase (103). Coenzyme Q10 is a promising supplement for preventing cardiovascular disease and treating heart failure patients (104). In summary, interventions targeting different nodes of ferroptosis (iron metabolism, antioxidant defense, LPO trapping) constitute a multi-layered pharmacological framework for atherosclerosis prevention and treatment, although clinical translation still requires more evidence.

### 5.2 Targeting pyroptosis

In the inflammatory process of atherosclerosis, targeting the upstream key node of pyroptosis—the NLRP3 inflammasome—has emerged as an effective intervention strategy. Preclinical studies have confirmed that the selective NLRP3 inhibitor MCC950 significantly suppresses plaque progression and improves stability in mouse models (105). The translational potential of this target is substantial, with multiple inhibitors having entered clinical development for inflammatory diseases (106). The most clinically impactful breakthrough comes from the classic anti-inflammatory drug colchicine, which functions by inhibiting NLRP3 assembly. Large-scale clinical trials have consistently demonstrated that low-dose colchicine reduces the risk of major adverse cardiovascular events by 23-31% in patients post-myocardial infarction or with chronic coronary disease (107, 108). Based on this high-level evidence, latest international guidelines have recommended its use for secondary prevention of atherosclerotic cardiovascular disease (109). Meanwhile, the discovery of novel inhibitors targeting the terminal execution step of pyroptosis (such as GSDMD pore formation), including necrosulfonamide, provides new directions for precisely inhibiting inflammatory cell death (110).

### 5.3 Dual targeting strategies

The complex interaction between ferroptosis and pyroptosis in atherosclerosis suggests that combined targeting may yield synergistic benefits. GSDMD, the key pyroptosis execution protein, can cause mitochondrial membrane permeabilization after pore formation, potentially exacerbating mitochondrial dysfunction and oxidative stress, thereby potentially aggravating ferroptosis (87). This provides a common node for intervention: inhibiting GSDMD not only blocks pyroptosis but may also indirectly counteract ferroptosis through derived mitochondrial protection. Disulfiram, the first identified small molecule inhibitor of GSDMD, blocks its pore formation through covalent modification, providing a tool drug for targeting this node (89). On the other hand, mitochondria themselves are core organelles regulating ferroptosis, and their dysfunction is a central feature of ferroptosis (111). Therefore, therapies protecting mitochondria hold promise for simultaneously mitigating both forms of cell death. Furthermore, some existing drugs have demonstrated multi-pathway benefits: statins, beyond their lipid-lowering effects, exert clinical anti-inflammatory effects partly attributable to inhibition of NLRP3 inflammasome activation (112, 113), which may simultaneously attenuate pyroptosis initiation. In summary, future therapeutic strategies could focus on developing combination therapies that simultaneously target key nodes of ferroptosis and pyroptosis to achieve more comprehensive intervention in atherosclerosis.

## 6 Conclusion and perspectives

OxLDL-induced ferroptosis and pyroptosis represent interconnected cell death mechanisms driving inflammation and plaque instability in atherosclerosis. The discovery of bidirectional crosstalk—GSDMD promoting LPO and ferroptotic DAMPs activating NLRP3—reveals a previously unrecognized positive feedback loop that amplifies vascular inflammation. This integrated perspective provides a mechanistic framework for understanding the persistent inflammatory component of atherosclerosis and identifies multiple therapeutic targets.

Several questions warrant future investigation. Single-cell technologies should be applied to map the spatiotemporal dynamics of ferroptosis and pyroptosis at different disease stages and plaque regions. Development of reliable biomarkers reflecting ferroptosis-pyroptosis axis activation will facilitate patient stratification for targeted therapies. Clinical trials evaluating dual targeting strategies will help determine whether combined pathway inhibition offers superior efficacy. The potential risks of immunosuppression from long-term cell death pathway inhibition need careful assessment in the context of chronic cardiovascular disease.

The ferroptosis-pyroptosis axis represents a promising therapeutic frontier for addressing residual inflammatory risk in atherosclerotic cardiovascular disease. Translating these mechanistic insights into clinical applications holds the potential to reduce the substantial disease burden that persists under current therapeutic approaches.
